# Functional diversity of urban bird communities: effects of landscape composition, green space area and vegetation cover

**DOI:** 10.1002/ece3.1778

**Published:** 2015-10-22

**Authors:** Claudia Schütz, Christian H. Schulze

**Affiliations:** ^1^ Department of Botany and Biodiversity Research University of Vienna Vienna Austria

**Keywords:** Avifaunal richness, ecological function, environmental filter, landscape composition, tree cover, urban birds, urban ecology, urban green area

## Abstract

In this study, we aim to gain a better insight on how habitat filtering due to urbanization shapes bird communities of Vienna city parks. This may help to derive implications for urban planning in order to promote and maintain high diversity and ecosystem function in an increasing urbanized environment. The structure of wintering bird communities of 36 Vienna city parks – surveyed once a month in January 2009, December 2009, December 2012, and January 2013 – was described by species richness and the functional diversity measurements FRic (functional richness), FEve (functional evenness), and FDiv (functional divergence). Environmental filtering was quantified by park size, canopy heterogeneity within the park, and the proportion of sealed area surrounding each park. Species richness, FRic, and FDiv increased with increasing park size. Sealed area had a strong negative effect on species richness and FDiv. Canopy heterogeneity played a minor role in explaining variance in FDiv data. FEve did not respond to any of these park parameters. Our results suggest a loss of species richness and functional diversity, hence most likely indicate a decline in ecosystem function, with decreasing park size and increasing sealed area of the surrounding urban landscape matrix.

## Introduction

As the human population is continuously growing, landscapes are increasingly affected by urbanization. In 2010, more than 50% of the world population inhabited urban areas, and by 2050, even 70% of the human population are expected to live in cities (UN 2012). This ongoing urban development leads to a fragmentation, isolation, and degradation of natural habitats, being accompanied by severe impacts on the biotic communities living in urban environments (Alberti 2005; McKinney 2006), such as arthropods (Bergerot et al. 2011; Vergnes et al. 2012), reptiles and amphibians (Hamer and McDonnell 2010), or small mammals (Gomes et al. 2011).

Numerous studies on urban bird communities have also already shown that urbanization can cause changes in community composition, a decrease in species richness, and a loss of species diversity (Marzluff 2001; Chace and Walsh 2006; Reis et al. 2012; Yu and Guo 2013; Ferenc et al. 2014). Urbanization acts as an environmental filter, resulting in higher functional similarity of bird community with increasing urbanization (Croci et al. 2008; Meffert and Dziock 2013; Sol et al. 2014). Although all these studies report a decrease in functional diversity due to urbanization, these results are heterogeneous, often lacking standard and well‐known indices (Filippi‐Codaccioni et al. 2009). Additionally, Mason et al. (2005) argued that functional diversity cannot be quantified by a single index. To consider the different facets of functional diversity, the indices FRic (functional richness), FEve (functional evenness), and FDiv (functional divergence) were proposed, as they are continuous, abundance based (FEve, FDiv), incorporate multiple functional traits, and are independent of species richness (FEve, FDiv) and of each other (Vílleger et al. 2008; Mouchet et al. 2010). FRic is used for quantifying the niche space filled by a community (Vílleger et al. 2008). Functional evenness describes the evenness of abundance distribution of species in a niche space (Vílleger et al. 2008). FDiv represents the abundance distribution within the functional trait space occupied by a community (Vílleger et al. 2008). The first studies testing how these different facets of functional diversity respond to habitat and landscape modification have already shown that habitat filtering can shape bird communities in fragmented systems (Filippi‐Codaccioni et al. 2009; Ding et al. 2013; Barbaro et al. 2014).

Birds are particularly suitable to analyze functional diversity patterns as there is comprehensive information available on their biological characteristics (e.g., Glutz von Blotzheim 1985–1999; Dunning 2008), which is essential when working with functional diversity measurements based on morphological, physiological, and behavioral traits.

In our study, environmental filtering will be characterized at two spatial scales. On a local scale, the city park characteristics park size and canopy heterogeneity will be considered. Park size has already been identified to be one of the most important factors influencing bird diversity and community composition in city parks with larger parks showing higher taxonomic and functional avian diversity than smaller ones due to an increase in habitat complexity (Jokimäki 1999; Fernández‐Juricic 2000a; Fernández‐Juricic and Jokimäki 2001). As in geographical regions naturally covered by forest, city parks with high amounts of woody vegetation are predominantly inhabited by forest birds and vegetation variables such as foliage cover, tree height, or area of woodlot often play an important role (Jokimäki 1999; MacGregor‐Fors 2008). In our study, canopy heterogeneity (perimeter of closed canopy/area of closed canopy) of city parks – as size‐independent vegetation variable – will be considered. A high degree of canopy heterogeneity indicates a higher fragmentation of tree cover within a city park and may influence functional diversity of bird communities, as species being habituated to human activities are known to be edge specialists, whereas species with specific habitat requirements depend on the interior areas of closed woodlot (Fernández‐Juricic 2001). On a landscape scale, we expect that environmental filtering will be driven by the urban matrix adjacent to each city park, which can modify connectivity of remaining green spaces. Landscape connectivity, defined as the “degree to which the landscapes facilitate or impede movement among resource patches” (Tischendorf and Fahrig 2000), can be a key factor for maintaining species diversity in fragmented landscapes (Martensen et al. 2008; Shanahan et al. 2011). As a result, also city parks surrounded by a less urbanized landscape show higher numbers of bird species and individuals, attracting especially woodland species, insectivores, and cavity nesting birds (Carbó‐Ramírez and Zuria 2011; Ikin et al. 2013).

This study examines how habitat filtering due to urbanization influences functional trait distributions and as a consequence structures bird communities of Vienna city parks. Therefore, species richness and the three functional diversity indices FRic, FEve, and FDiv will be used to gain a better insight on the influence of environmental filters shaping bird communities of human‐dominated landscapes (Vílleger et al. 2008). In accordance with the results of other studies (Reis et al. 2012; Yu and Guo 2013), we also expect a loss of species diversity with increasing urbanization. Furthermore, we predict a decrease in FRic with increasing urbanization, due to increased functional similarity of bird species in highly urbanized areas (Luck and Smallbone 2011; Meffert and Dziock 2013). Birds vary in their ability to adapt to changes along the urban–rural gradient, with urbanization filtering for species based on their biological traits (McKinney 2002; Chace and Walsh 2006). Hence, with increasing urbanization, we expect a functionally more concentrated distribution of species within functional space, leading to a decrease in FDiv. A few dominant species possess superior abilities for living in urban environments, become even dependent on urban resources, and peak in their abundances in the urban core area (McKinney 2002). Therefore, we expect a decrease in FEve with a higher degree of urbanization.

## Materials and Methods

### Study area

**Figure 1:**
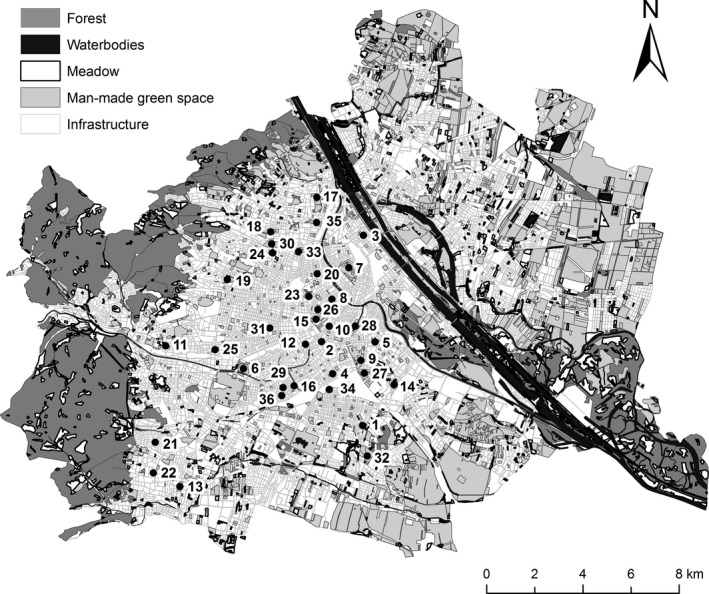
Overview on 36 city parks of Vienna where bird surveys were carried out. Black circles indicate the midpoint of each city park; city park codes refer to Table S1.

The study was carried out in Vienna (48°13′ N, 16°22′ E), the capital city of Austria, located at the northeastern extension of the Alps and predominantly situated within the Vienna Basin (Berger and Ehrendorfer 2011). A total of 208.42 km^2^ (50.2%) of the city area is not built‐up. Nearly 6% consist of city parks and other man‐made green space. Bird communities were assessed in 36 city parks, ranging from 0.4 ha to 34.5 ha in size and spread across the built‐up area of Vienna, southwest of the river Danube (Fig. 1).

### Quantification of city park habitat variables and urban matrix

For each city park, area and tree cover were considered. Park size was calculated in ArcGIS 10.0 (ESRI, Redlands, CA, USA) based on Vienna land use data of the year 2009 (Table S1). Tree cover of each city park was digitized in ArcGIS 10.0 (ESRI) using satellite images of the map service “ArcGIS Online basemaps” (0.3‐m spatial resolution, date of origin: August 2011). For calculating the canopy cover heterogeneity of each city park, the perimeter of digitized canopy (m) was divided by the area of closed canopy (m^2^), defined as closed leaf cover tolerating gaps up to 5 m (Table S1).

The urban landscape surrounding each city park was described based on Vienna land use data from the period 2010 to 2012. In ArcGIS 10.0 (ESRI), a circle with a radius of 500 m – centered on the centroid of each park – was clipped out of the land use shape file. Then, the 55 categories of the land use data were simplified to four categories, describing the permeability of the landscape for avifauna: natural green space (e.g., pasture), man‐made green space (e.g., lawn, meadow, and other unsealed areas), sealed areas (e.g., roads, buildings), and forest/tree‐covered areas. For each land use category, the area within the circle was calculated, excluding the park area. The proportion of the park area within the circle of radius 500 m varied between 0.5% for the smallest park (Börsepark: 0.4 ha) and 44% for the largest park (Augarten: 34.5 ha).

### Bird surveys

Available data on wintering bird communities of Vienna city parks – covering 3 years – were used. Bird surveys were carried out by groups averagely consisting of at least one experienced field ornithologist, being assisted by three further observers. Surveys were conducted once a month in January 2009, December 2009, December 2012, and January 2013. Each park was surveyed between 08:00 AM and 15:30 PM under good weather conditions (i.e., avoiding windy days and/or days of heavy rain and snowfall, respectively). Sampling effort was standardized according to park size (10 min per 1 ha). The existing road network within a park was used for survey routes, trying to cover the entire area of the park in a zigzag manner. Carrying out surveys from existing roads may cause samples unrepresentative of the surrounding area because of greater disturbance and presence of “edge habitats” close to the roads (Buckland et al. 2008). However, the density of roads and paths in the surveyed Vienna city parks is rather high and our surveys were conducted during the winter, when the majority of trees are without leaves. Hence, we think that most of the bird species could be detected and neither using path‐based transects nor varying detectability of bird species was a major source of bias. All species and the number of birds heard or seen were recorded (except overflying birds), avoiding double‐counting as effectively as possible (Table S2). Waterfowl and birds with a strong affiliation to water (e.g., Grey Wagtail *Motacilla cinerea*) were excluded from further analyses as their occurrence is strongly driven by the presence of suitable waterbodies.

### Quantification of avian functional diversity

Functional diversity was quantified by using four measurements. For each city park, species richness, indicating taxonomic diversity, was quantified as the total number of observed species in the survey months January 2009, December 2009, December 2012, and January 2013.

Furthermore, three functional diversity metrics were used. For measuring FRic, quantifying the volume of functional trait space occupied, the convex hull volume was calculated, using the Quickhull algorithm (Barber et al. 1996; Vílleger et al. 2008). Basically, this convex hull algorithm determines the most extreme trait values, links them to build the convex hull, and calculates the volume of this convex hull (Vílleger et al. 2008). For calculating FEve, a minimum spanning tree is used that links all the points in a multidimensional trait space with the minimum sum of branch lengths (Vílleger et al. 2008). Functional evenness then measures the regularity of points along this tree and the regularity in their abundances (Vílleger et al. 2008). FDiv is measured using an index that quantifies how species differ in their distances (weighted by their abundances) from the center of gravity in the functional space (Vílleger et al. 2008).

For calculating functional diversity indices, a trait matrix was built. Twenty functional traits were selected, which are commonly used in functional diversity research of bird communities (Petchey et al. 2007; Flynn et al. 2009; Ding et al. 2013), describing how organisms acquire resources from their environment and reflecting resource use requirements (Tables 1, S3). Data on the trait categories foraging substrate, foraging method, and diet were extracted from faunal monographs (Glutz von Blotzheim 1985–1999;). Data on body mass were extracted from Dunning (2008). The functional diversity metrics were calculated in R 2.15.1 (R Core Team 2012), using the function dbFD of the FD package (Laliberté and Legendre 2010; Laliberté and Shipley 2011).

**Table 1:** Functional traits used for calculating functional diversity indices of wintering bird communities in Vienna city parks

Trait category	Trait	Type of variable	Range or short description of categories
Resource quantity	Body mass [g]	Continuous	6.8–1246.8
Foraging substrate	Ground	Categorical	0 = not used
	Foliage		1 = rarely used
	Bark		2 = moderately used
	Air		3 = often used
Foraging method	Gleaning	Categorical	0 = not used
	Pecking		1 = rarely used
	Hawking		2 = moderately used
	Sally		3 = often used
	Probing		
Diet	Mammals	Categorical	
	Fishes		
	Amphibians, reptiles		
	Birds		0 = not used
	Carrion		1 = rarely used
	Arthropods		2 = moderately used
	Annelids		3 = often used
	Snails		
	Fruits		
	Seeds		

### Statistical analysis

Due to strong multicollinearity of urban landscape variables, we selected the variable sealed area as proxy for landscape permeability (Table S4). An increasing fraction of sealed areas proved to negatively affect bird species richness in urban areas (Fontana et al. 2011).

Multiple linear regressions were carried out to describe relationships between city park variables and taxonomical and functional diversity measures of bird communities. In advance, park size was log‐transformed (log *x* + 1) to improve the fit to normality. Models were fitted using all predictor variables and possible subsets. To identify predictor variables with the strongest influence on the response variables, models were ranked according to their information content determined by the Akaike's information criterion corrected for small‐sample bias (AIC_c_) as the sample size divided by the number of parameters included in the models was <40 (Symonds and Moussalli 2011). Best models, having lowest AIC_c_ values and thus highest Akaike weights (as the relative likelihood of the model being the best), were considered to have the best fit with the data. Models that had an AIC_c_ difference (∆_*i*_) <2 from the best model were considered to be “best ranked.” To describe the effects of the variables that affected the functional diversity measures after controlling for other variables, *β* coefficients were used. Furthermore, predicted relationships between avian diversity measures and the park characteristics included in the models with the lowest AIC_c_ value were plotted. All statistical analyses were carried out using the R packages AICcmodavg, rms, and QuantPsyc.

## Results

**Figure 2:**
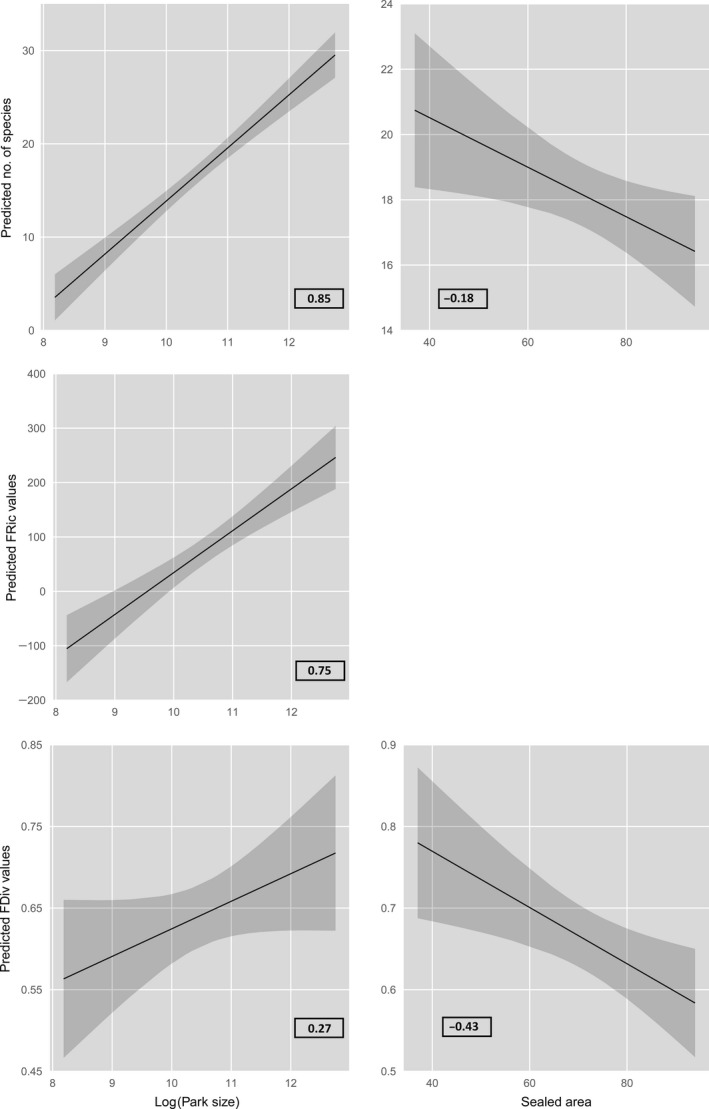
Predicted relationships between the two city park characteristics park size (ha) and proportion of sealed area (%) included in the model with the lowest AIC_c_ value and no. of species (taxonomic diversity), FRic (functional richness), and FDiv (functional divergence). Correlation coefficients (*β* coefficients) of each relationship are listed in the graphs.

Park size was included in four of the six best ranked models of taxonomical and functional diversity metrics and positively affected species richness, FRic, and FDiv (Fig. 2, Table 2). Sealed area was part of the best ranked models for species richness and FDiv and had strong negative effects on these two bird metrics (Fig. 2, Table 2). Canopy heterogeneity was not included in models with lowest AIC_c_, but played a minor role in the best ranked models of FDiv (Table 2). None of the three predictor variables park size, sealed area, and canopy heterogeneity achieved a significant model fitting for the response variable FEve.

**Table 2:** Best ranked models (∆_*i*_ <2) for the bird metrics No. Spec. (species richness), FRic (functional richness), and FDiv (functional divergence). For all included variables, *β* coefficients are provided. Furthermore, number of estimable parameters (*K*), Akaike's information criterion corrected for small‐sample bias (AIC_c_), differences in AIC_c_ values of each model compared with the model with the lowest AIC_c_ value (∆_*i*_), and the Akaike weights (*w*_*i*_) are listed

	No. Spec.	FRic	FDiv			
	1.	1.	1.	2.	3.	4.
Variables included						
Park size	0.85	0.75	0.27		0.23	
Sealed area	−0.18		−0.43	−0.45	−0.39	−0.52
Canopy heterogeneity				−0.25	−0.21	
Model summary						
*K*	4	3	4	4	5	3
AIC_c_	183.10	415.04	−50.13	−49.71	−49.51	−49.36
∆_*i*_	0.00	0.00	0.00	0.42	0.62	0.76
*w*_*i*_	0.68	0.61	0.29	0.24	0.21	0.20
*R* ^2^	0.85	0.56	0.33	0.32	0.37	0.27
Adjusted *R* ^2^	0.84	0.54	0.29	0.28	0.31	0.24

## Discussion

### Park size

Park size is a very important parameter in explaining differences between city parks in the considered taxonomic and functional diversity metrics, as it was included in the best ranked models for species richness, FRic, and FDiv. As in other studies, park size strongly positively affected species richness (e.g., Jokimäki 1999; Fernández‐Juricic 2000a; Fernández‐Juricic and Jokimäki 2001; Murgui 2007). Even in very small urban green spaces, ranging from 0.1 to 2 ha in size, the area of green space was the most important variable positively influencing bird species richness in the city of Pachuca, Mexico (Carbó‐Ramírez and Zuria 2011). This positive species–area relationship also follows the predictions of the theory of island biogeography (MacArthur and Wilson 1967), which is often applied in the study of urban bird communities as urban parks represent remnant habitat patches being isolated from the surrounding urban matrix and therefore representing the only refuges for many bird species (Davis and Glick 1978; Fernández‐Juricic and Jokimäki 2001). Higher species numbers in larger parks may partly be explained by an increase in habitat complexity and resource availability in larger parks compared to smaller ones (Fernández‐Juricic and Jokimäki 2001; Cornelis and Hermy 2004). Hence, the specific requirements of certain forest or insectivorous bird species will be only met in larger habitat fragments (Fernández‐Juricic 2000a; Zanette et al. 2000).

Furthermore, large city parks – as remnants of seminatural green space embedded in an urban landscape – have larger core areas that are unaffected by effects associated with habitat edges, such as microclimatic differences, a higher predation risk, or higher levels of human disturbances (Saunders et al. 1991; Fernández‐Juricic 2001; Schneider et al. 2012). Therefore, in large parks beside edge specialist species, being highly habituated to human activities and showing high breeding densities at urban park edges, also species with specific habitat requirements can be found in the more undisturbed core areas (Fernández‐Juricic 2001).

Because of a greater habitat complexity and more prominent core areas in larger parks, increasing park size results not only in a higher number of recorded species, but also in an increase in FRic as larger niche space can be filled by avian communities. This is consistent with the results of a study on bird communities of land‐bridge islands in the Thousand Island Lake, China, where FRic was positively related to island area (Ding et al. 2013).

Park area was also positively related to FDiv, what may indicate a relaxation of environmental filters as emphasized for fish assemblages (Teresa and Casatti 2012). As FDiv increases with park size, bird communities in small parks show a functionally more concentrated distribution of species within functional space compared to communities of larger city parks (Mason et al. 2005). Small city parks have higher perimeter–core ratios than large ones, making them more open to edge effects associated with the urban matrix, such as higher levels of car and pedestrian traffic (Fernández‐Juricic 2001). As a consequence, the environmental filtering due to urbanization may be enhanced, limiting the occurrence of high trait diversity and resulting in highly similar communities (Teresa and Casatti 2012). These communities may mainly consist of urban exploiters, which are highly adapted to urban environments, as they are dependent on human resources and are independent of amount and structure of vegetation (Blair 2001; McKinney 2008). In contrast, large habitat patches are characterized by an increase in habitat complexity and availability of resources such as food and nest sites (Saunders et al. 1991; Cornelis and Hermy 2004; Sitompul et al. 2004). Therefore, large city parks provide a large diversity of habitats necessary to support many species with different habitat requirements (Fernández‐Juricic and Jokimäki 2001).

### Sealed area

In accordance with other studies on bird communities of urban green spaces, the increasing urbanization degree of the surrounding landscape negatively affected species richness of city parks (Carbó‐Ramírez and Zuria 2011; Ikin et al. 2013). Forest bird species moving through fragmented landscapes are strongly limited by the degree of patch connectivity, quantified by the presence of corridors or the distance between patches (Norris and Stutchbury 2001; Uezu et al. 2005). As urban bird populations in city parks can be seen as sets of semi‐independent populations embedded in an inhospitable urban matrix connected by dispersal to themselves and to regional populations (Fernández‐Juricic 2004), they may also be affected by the permeability of the urban matrix. Therefore, an increase in urbanization degree reduces the permeability of the urban landscape due to longer distances between remnant habitat patches and lower number of corridors such as wooded streets, both exerting a positive influence on the connectivity between city parks (Fernández‐Juricic 2000b; Husté and Boulinier 2011). As a consequence, the dispersal abilities of habitat specialist species, reluctant to move through unsuitable urban matrix, may decrease, reducing the chances of park occupation and therefore local bird diversity (Fernández‐Juricic 2000a).

These decreasing dispersal abilities of habitat specialist species may also cause lower FDiv values of avian communities, indicating that city parks surrounded by high proportion of sealed area harbor bird species which are functionally more similar to each other (Teresa and Casatti 2012).

### Canopy heterogeneity

The extent of the tree layer within a city park is, especially for specialist bird species, of high importance as it provides nesting habitat, food resources, and refuge from predators and human disturbance (Murgui 2007). Consequently, bird species richness in urban areas is correlated with tree density (Sandström et al. 2006). A decrease in closed forest cover confronts birds with a higher degree of edge habitats and the negative effects linked to it, such as an increase in predation risk or human disturbance (Fernández‐Juricic 2001; Schneider et al. 2012). In contrast to other studies, showing effects of forest edges on the considered bird responses (Barbaro et al. 2014), canopy heterogeneity played a minor role in explaining the taxonomical and functional diversity indices, as it was not included in the models with the lowest AIC_c_ values.

## Conclusion

Surprisingly, FEve did not respond to changes in park characteristics, although it proved to be sensitive to environmental filtering in simulated communities (Mouchet et al. 2010). Also empirical data showed that FEve was negatively affected by habitat fragmentation and environmental gradients of disturbance, respectively (Filippi‐Codaccioni et al. 2009; Pakeman 2011; Ding et al. 2013). In contrast, the declines of FRic and FDiv provide strong evidence for a loss of functional diversity from small toward large city parks. This indicates that bird assemblages of parks embedded in an urban landscape matrix with a high permeability for forest birds (due to a high density of green spaces) most likely provide an increased ecosystem function. These results have important implications for urban planning aiming to promote and maintain high diversity and ecosystem function in modern human‐dominated landscapes.

## Conflict of Interest

None declared.

## Supporting information


**Table S1.** Habitat characteristics of 36 Vienna city parks, where winter bird surveys were conducted. Beside park size and canopy heterogeneity, also the area of each habitat parameter describing the urban landscape matrix in a circle of radius 500 m around the centroid of each city park are presented. Landscape measures used for analyses are shaded in grey. Also calculation of canopy heterogeneity and proportion of sealed area is indicated.

**Table S2.** Total number of individuals of bird species recorded in 36 city parks in Vienna.

**Table S3.** Biological traits of all bird species considered in this study.

**Table S4.** Correlation coefficients of Pearson correlations including the park variables park area (log transformed) and canopy heterogeneity as well as the landscape variables natural green space, man‐made green space, forest and sealed area, describing the urban matrix surrounding each city park, are listed. Significant correlations (*P* < 0.05) are printed in bold.
