# Supplementary material for: Functional diversity of urban bird communities: effects of landscape composition, green space area and vegetation cover
Source: Ecol Evol. 2015 Oct 22;5(22):5230–9. doi: 10.1002/ece3.1778 (PMC6102532; doi:10.1002/ece3.1778)
Supplement: Supplementary file 1 — Table S1. Habitat characteristics of 36 Vienna city parks, where winter bird surveys were conducted. Beside park size and canopy heterogeneity, also the area of each habitat parameter describing the urban landscape matrix in a circle of radius 500 m around the centroid of each city park are presented. Landscape measures used for analyses are shaded in grey. Also calculation of canopy heterogeneity and proportion of sealed area is indicated. [file ECE3-5-5230-s001.docx]

**Table S1:** Habitat characteristics of 36 Vienna city parks, where winter bird surveys were conducted. Beside park size and canopy heterogeneity, also the area of each habitat parameter describing the urban landscape matrix in a circle of radius 500 m around the centroid of each city park are presented. Landscape measures used for analyses are shaded in grey. Also calculation of canopy heterogeneity and proportion of sealed area is indicated.

| **(V1) City park code** | **(V2) Urban park** | **(V3) Area (ha)** | **(V4) Canopy edge length (m)** | **(V5) Closed canopy area (m^2^)** | **(V6) Canopy heterogeneity (m/ m^2^) [= V4/V5]** | **(V7) Natural green space (m^2^)** | **(V8) Man-made green space (m^2^)** | **(V9) Forest (m^2^)** | **(V10) Sealed area (m^2^)** | **(V11) Proportion of sealed area**  **[=V10/(V7+V8+V9+V10)]** |
| --- | --- | --- | --- | --- | --- | --- | --- | --- | --- | --- |
| 01 | Alfred Böhm Park | 2.8 | 1204.06 | 17816.80 | 0.068 | 30961.70 | 206679.72 | 0.00 | 490564.12 | 0.67 |
| 02 | Alfred-Grünwald-Park | 0.8 | 694.60 | 3149.60 | 0.221 | 0.00 | 58616.13 | 0.00 | 714901.05 | 0.92 |
| 03 | Allerheiligenpark | 2.1 | 1257.11 | 13595.00 | 0.092 | 43215.60 | 97159.12 | 0.00 | 540368.86 | 0.79 |
| 04 | Alois Drasche Park | 2.0 | 1471.30 | 12709.70 | 0.116 | 3.97 | 99267.47 | 0.00 | 596500.12 | 0.86 |
| 05 | Arenbergpark | 2.9 | 1670.61 | 15744.50 | 0.106 | 193.40 | 122001.92 | 0.00 | 619418.00 | 0.84 |
| 06 | Auerwelsbachpark | 14.2 | 10250.50 | 63300.70 | 0.162 | 39433.00 | 130396.24 | 0.00 | 413268.25 | 0.71 |
| 07 | Augarten | 34.5 | 20239.70 | 186473.00 | 0.109 | 17663.00 | 89770.62 | 0.00 | 320874.03 | 0.75 |
| 08 | Börsepark | 0.4 | 248.59 | 2622.71 | 0.095 | 0.00 | 45564.42 | 0.00 | 711327.13 | 0.94 |
| 09 | Botanischer Garten | 9.6 | 4791.21 | 52391.10 | 0.091 | 73450.00 | 109615.97 | 0.00 | 479202.65 | 0.72 |
| 10 | Burggarten | 2.9 | 1787.13 | 10538.90 | 0.170 | 0.00 | 89294.07 | 0.00 | 659930.98 | 0.88 |
| 11 | Casinopark Baumgarten | 4.3 | 2572.62 | 17952.20 | 0.143 | 27008.00 | 289974.23 | 0.00 | 355918.74 | 0.53 |
| 12 | Esterhazypark | 1.0 | 979.30 | 5289.63 | 0.185 | 2086.47 | 62416.08 | 0.00 | 704840.25 | 0.92 |
| 13 | Fridtjof-Nansen-Park | 6.6 | 3838.70 | 26215.40 | 0.146 | 18416.30 | 324555.18 | 0.00 | 345180.16 | 0.50 |
| 14 | Friedhof St. Marx | 6.7 | 1234.78 | 60226.60 | 0.021 | 30830.43 | 157479.61 | 174.40 | 479679.01 | 0.72 |
| 15 | Grete Rehor Park | 0.7 | 627.37 | 3427.20 | 0.183 | 0.00 | 133840.15 | 0.00 | 632979.64 | 0.83 |
| 16 | Haydnpark | 2.1 | 787.05 | 11662.90 | 0.067 | 26517.10 | 97188.90 | 0.00 | 595374.13 | 0.83 |
| 17 | Heiligenstädterpark | 8.0 | 4340.36 | 53221.70 | 0.082 | 2024.40 | 361010.63 | 0.00 | 310188.23 | 0.46 |
| 18 | Hugo-Wolf-Park | 5.9 | 2992.76 | 40171.00 | 0.075 | 45445.40 | 239877.88 | 27573.90 | 404195.12 | 0.56 |
| 19 | Kongresspark | 4.6 | 3304.66 | 23434.40 | 0.141 | 0.00 | 188660.10 | 0.00 | 517416.36 | 0.73 |
| 20 | Liechtensteinpark | 4.8 | 2236.28 | 23095.00 | 0.097 | 0.00 | 94885.86 | 0.00 | 629103.70 | 0.87 |
| 21 | Napoleonwald | 3.1 | 1885.16 | 19762.80 | 0.095 | 4210.90 | 459655.10 | 0.00 | 272321.42 | 0.37 |
| 22 | Ölzeltpark | 1.1 | 569.92 | 7755.72 | 0.073 | 11179.07 | 408339.13 | 19605.80 | 326488.13 | 0.43 |
| 23 | Ostarrichipark | 1.1 | 308.92 | 2342.54 | 0.132 | 0.00 | 126929.19 | 0.00 | 638491.98 | 0.83 |
| 24 | Park der Universitätssternwarte | 5.8 | 631.57 | 52134.10 | 0.012 | 8400.50 | 159671.00 | 0.30 | 520933.09 | 0.76 |
| 25 | Penzinger Friedhof | 4.5 | 2750.19 | 26800.90 | 0.103 | 0.00 | 94939.66 | 0.00 | 634550.59 | 0.87 |
| 26 | Rathauspark | 4.5 | 2558.90 | 27561.30 | 0.093 | 23738.00 | 126517.95 | 0.00 | 313726.34 | 0.68 |
| 27 | Schweizergarten | 15.9 | 8266.24 | 81700.50 | 0.101 | 0.00 | 50220.17 | 0.00 | 565850.96 | 0.92 |
| 28 | Stadtpark | 13.4 | 7929.05 | 63392.00 | 0.125 | 13218.40 | 122195.24 | 0.00 | 631507.62 | 0.82 |
| 29 | Steinbauerpark | 1.1 | 446.29 | 5153.12 | 0.087 | 92531.50 | 220609.20 | 51955.60 | 395035.73 | 0.52 |
| 30 | Türkenschanzpark | 15.5 | 10301.30 | 95932.00 | 0.107 | 23045.00 | 231694.13 | 57979.66 | 313604.69 | 0.50 |
| 31 | Vogelweidpark | 1.8 | 1138.59 | 7495.30 | 0.152 | 0.00 | 86557.49 | 0.00 | 671276.98 | 0.89 |
| 32 | Volkspark | 19.2 | 10226.30 | 100487.00 | 0.102 | 78971.00 | 256822.15 | 1331.06 | 246802.22 | 0.42 |
| 33 | Währingerpark | 6.8 | 4216.33 | 33891.60 | 0.124 | 18892.50 | 197568.81 | 0.00 | 488160.01 | 0.69 |
| 34 | Waldmüllerpark | 4.4 | 2215.72 | 30833.10 | 0.072 | 0.00 | 78664.11 | 15874.10 | 559790.28 | 0.86 |
| 35 | Wertheimsteinpark | 6.8 | 4443.41 | 44196.50 | 0.101 | 18457.20 | 210613.88 | 332.51 | 417508.81 | 0.65 |
| 36 | Wilhelmsdorfer Park | 2.2 | 1298.82 | 9591.59 | 0.135 | 0.00 | 128424.60 | 5289.31 | 555194.28 | 0.81 |
