# Supplementary material for: Functional diversity of urban bird communities: effects of landscape composition, green space area and vegetation cover
Source: Ecol Evol. 2015 Oct 22;5(22):5230–9. doi: 10.1002/ece3.1778 (PMC6102532; doi:10.1002/ece3.1778)
Supplement: Supplementary file 2 — Table S2. Total number of individuals of bird species recorded in 36 city parks in Vienna. [file ECE3-5-5230-s002.docx]

**Table S2:** Total number of individuals of bird species recorded in 36 city parks in Vienna. City park codes in the columns refer to Table S1.

| **Species** | **1** | **2** | **3** | **4** | **5** | **6** | **7** | **8** | **9** | **10** | **11** | **12** | **13** | **14** | **15** | **16** | **17** | **18** | **19** | **20** | **21** | **22** | **23** | **24** | **25** | **26** | **27** | **28** | **29** | **30** | **31** | **32** | **33** | **34** | **35** | **36** |
| --- | --- | --- | --- | --- | --- | --- | --- | --- | --- | --- | --- | --- | --- | --- | --- | --- | --- | --- | --- | --- | --- | --- | --- | --- | --- | --- | --- | --- | --- | --- | --- | --- | --- | --- | --- | --- |
| *Accipiter nisus* | 0 | 0 | 0 | 0 | 0 | 0 | 1 | 0 | 1 | 0 | 1 | 0 | 0 | 1 | 0 | 0 | 0 | 0 | 0 | 0 | 0 | 0 | 0 | 0 | 0 | 0 | 1 | 0 | 0 | 1 | 0 | 0 | 0 | 1 | 2 | 0 |
| *Aegithalos caudatus* | 0 | 0 | 0 | 0 | 0 | 0 | 0 | 0 | 0 | 0 | 0 | 0 | 0 | 23 | 0 | 0 | 4 | 0 | 0 | 0 | 0 | 0 | 0 | 9 | 0 | 0 | 0 | 0 | 0 | 0 | 0 | 0 | 0 | 0 | 0 | 0 |
| *Bombycilla garrulus* | 230 | 0 | 0 | 0 | 0 | 0 | 11 | 0 | 0 | 0 | 0 | 0 | 130 | 0 | 0 | 0 | 30 | 0 | 0 | 0 | 0 | 0 | 0 | 0 | 40 | 9 | 194 | 0 | 0 | 124 | 0 | 1 | 380 | 0 | 60 | 0 |
| *Buteo buteo* | 0 | 0 | 0 | 0 | 0 | 0 | 0 | 0 | 0 | 0 | 0 | 0 | 0 | 0 | 0 | 0 | 0 | 0 | 0 | 0 | 0 | 0 | 0 | 0 | 0 | 0 | 0 | 0 | 0 | 0 | 0 | 1 | 0 | 0 | 0 | 0 |
| *Carduelis carduelis* | 0 | 0 | 1 | 51 | 19 | 0 | 16 | 11 | 16 | 0 | 1 | 0 | 55 | 0 | 0 | 0 | 9 | 0 | 10 | 44 | 15 | 22 | 0 | 0 | 5 | 5 | 4 | 11 | 1 | 12 | 5 | 22 | 0 | 1 | 0 | 12 |
| *Carduelis spinus* | 0 | 0 | 0 | 0 | 0 | 0 | 0 | 0 | 0 | 0 | 0 | 0 | 0 | 0 | 0 | 0 | 0 | 0 | 0 | 0 | 1 | 0 | 0 | 0 | 1 | 0 | 1 | 1 | 0 | 5 | 0 | 1 | 0 | 0 | 0 | 0 |
| *Certhia brachydactyla* | 0 | 0 | 0 | 0 | 0 | 1 | 1 | 0 | 0 | 0 | 0 | 0 | 0 | 0 | 0 | 0 | 2 | 1 | 0 | 0 | 2 | 0 | 0 | 1 | 3 | 0 | 4 | 0 | 0 | 2 | 0 | 1 | 0 | 0 | 2 | 0 |
| *Certhia familiaris* | 0 | 0 | 0 | 0 | 0 | 0 | 0 | 0 | 1 | 1 | 1 | 0 | 0 | 0 | 0 | 1 | 1 | 0 | 0 | 0 | 0 | 0 | 0 | 0 | 2 | 0 | 8 | 0 | 0 | 3 | 0 | 3 | 0 | 0 | 0 | 0 |
| *Chloris chloris* | 0 | 2 | 0 | 6 | 30 | 25 | 148 | 1 | 27 | 5 | 40 | 1 | 42 | 40 | 0 | 0 | 3 | 10 | 36 | 3 | 29 | 1 | 0 | 9 | 18 | 30 | 102 | 83 | 1 | 9 | 6 | 31 | 12 | 14 | 5 | 1 |
| *Coccothraustes coccothraustes* | 2 | 0 | 0 | 0 | 0 | 3 | 6 | 0 | 1 | 0 | 7 | 0 | 9 | 0 | 0 | 0 | 33 | 6 | 13 | 0 | 6 | 4 | 0 | 17 | 2 | 0 | 32 | 11 | 0 | 26 | 0 | 1 | 5 | 16 | 14 | 0 |
| *Columba livia f. domestica* | 58 | 20 | 444 | 157 | 192 | 132 | 808 | 45 | 10 | 132 | 0 | 201 | 3 | 6 | 158 | 106 | 12 | 0 | 91 | 1 | 0 | 0 | 23 | 0 | 121 | 111 | 962 | 572 | 401 | 102 | 47 | 29 | 48 | 87 | 50 | 457 |
| *Columba palumbus* | 0 | 0 | 0 | 0 | 0 | 5 | 0 | 0 | 0 | 0 | 0 | 0 | 0 | 0 | 0 | 0 | 9 | 2 | 0 | 0 | 30 | 0 | 0 | 0 | 0 | 0 | 0 | 0 | 0 | 9 | 0 | 1 | 0 | 0 | 0 | 0 |
| *Corvus corone* | 16 | 0 | 8 | 21 | 106 | 141 | 516 | 0 | 66 | 42 | 12 | 5 | 10 | 9 | 8 | 10 | 95 | 28 | 62 | 31 | 14 | 0 | 2 | 19 | 6 | 23 | 153 | 141 | 4 | 235 | 2 | 106 | 79 | 2 | 11 | 4 |
| *Corvus frugilegus* | 156 | 1 | 5 | 58 | 163 | 156 | 207 | 0 | 55 | 36 | 62 | 4 | 304 | 46 | 41 | 37 | 115 | 269 | 121 | 3 | 100 | 236 | 24 | 0 | 273 | 42 | 240 | 84 | 3 | 477 | 2 | 484 | 144 | 140 | 27 | 18 |
| *Corvus monedula* | 0 | 0 | 0 | 0 | 0 | 0 | 23 | 0 | 0 | 0 | 0 | 0 | 0 | 0 | 0 | 0 | 0 | 0 | 0 | 0 | 2 | 0 | 0 | 0 | 0 | 0 | 0 | 0 | 0 | 0 | 0 | 0 | 0 | 2 | 0 | 0 |
| *Cyanistes caeruleus* | 24 | 7 | 7 | 19 | 31 | 61 | 101 | 0 | 43 | 14 | 12 | 4 | 13 | 34 | 1 | 7 | 38 | 14 | 23 | 27 | 11 | 8 | 1 | 48 | 32 | 14 | 33 | 86 | 6 | 55 | 4 | 62 | 36 | 30 | 29 | 4 |
| *Dendrocopos major* | 6 | 0 | 2 | 2 | 6 | 14 | 36 | 0 | 7 | 3 | 10 | 1 | 2 | 4 | 1 | 1 | 12 | 6 | 6 | 11 | 10 | 2 | 0 | 12 | 11 | 1 | 14 | 18 | 0 | 23 | 0 | 18 | 6 | 3 | 10 | 3 |
| *Dendrocopos medius* | 0 | 0 | 1 | 0 | 0 | 1 | 5 | 0 | 2 | 2 | 1 | 0 | 0 | 0 | 0 | 0 | 1 | 0 | 0 | 1 | 1 | 0 | 0 | 1 | 1 | 1 | 3 | 1 | 0 | 6 | 0 | 3 | 2 | 0 | 0 | 0 |
| *Emberiza citrinella* | 0 | 0 | 0 | 0 | 0 | 0 | 0 | 0 | 0 | 0 | 0 | 0 | 11 | 0 | 0 | 0 | 0 | 0 | 0 | 0 | 0 | 0 | 0 | 0 | 0 | 0 | 0 | 0 | 0 | 0 | 0 | 0 | 0 | 0 | 0 | 0 |
| *Erithacus rubecula* | 1 | 1 | 1 | 1 | 0 | 1 | 3 | 0 | 7 | 1 | 1 | 0 | 4 | 5 | 0 | 0 | 8 | 1 | 0 | 1 | 0 | 0 | 1 | 1 | 1 | 1 | 3 | 4 | 0 | 6 | 0 | 0 | 3 | 0 | 6 | 0 |
| *Falco peregrinus* | 0 | 0 | 0 | 0 | 0 | 0 | 1 | 0 | 0 | 0 | 0 | 0 | 0 | 0 | 0 | 0 | 0 | 0 | 0 | 0 | 0 | 0 | 0 | 0 | 0 | 0 | 0 | 0 | 0 | 0 | 0 | 0 | 0 | 0 | 0 | 0 |
| *Falco tinnunculus* | 0 | 0 | 0 | 0 | 0 | 0 | 0 | 0 | 0 | 0 | 0 | 0 | 0 | 0 | 0 | 0 | 0 | 0 | 0 | 1 | 0 | 0 | 0 | 0 | 0 | 0 | 0 | 0 | 0 | 0 | 0 | 1 | 0 | 0 | 0 | 0 |
| *Fringilla coelebs* | 5 | 0 | 0 | 0 | 3 | 25 | 33 | 0 | 10 | 5 | 24 | 0 | 12 | 88 | 0 | 0 | 25 | 2 | 20 | 12 | 4 | 2 | 0 | 13 | 16 | 13 | 13 | 20 | 0 | 33 | 0 | 54 | 19 | 9 | 15 | 2 |
| *Fringilla montifringilla* | 0 | 0 | 0 | 0 | 0 | 0 | 0 | 0 | 0 | 0 | 0 | 0 | 0 | 0 | 0 | 0 | 5 | 1 | 0 | 0 | 0 | 0 | 0 | 0 | 0 | 0 | 0 | 0 | 0 | 0 | 0 | 0 | 0 | 0 | 0 | 0 |
| *Garrulus glandarius* | 0 | 0 | 0 | 0 | 0 | 0 | 0 | 0 | 1 | 0 | 0 | 0 | 0 | 0 | 0 | 0 | 0 | 1 | 0 | 0 | 0 | 0 | 0 | 1 | 0 | 0 | 0 | 0 | 0 | 0 | 0 | 9 | 0 | 0 | 1 | 0 |
| *Lophophanes cristatus* | 0 | 0 | 0 | 0 | 0 | 0 | 0 | 0 | 0 | 0 | 0 | 0 | 3 | 0 | 0 | 0 | 0 | 1 | 0 | 0 | 0 | 0 | 0 | 0 | 0 | 0 | 0 | 0 | 0 | 3 | 0 | 0 | 0 | 0 | 0 | 0 |
| *Nucifraga caryocatactes* | 0 | 0 | 0 | 0 | 0 | 0 | 0 | 0 | 0 | 0 | 0 | 0 | 0 | 1 | 0 | 0 | 0 | 0 | 0 | 0 | 0 | 0 | 0 | 0 | 0 | 0 | 0 | 0 | 0 | 0 | 0 | 0 | 0 | 0 | 0 | 0 |
| *Parus major* | 103 | 41 | 31 | 58 | 103 | 201 | 642 | 12 | 169 | 74 | 74 | 23 | 81 | 140 | 9 | 48 | 231 | 64 | 118 | 88 | 26 | 29 | 7 | 150 | 110 | 63 | 143 | 244 | 8 | 223 | 28 | 277 | 81 | 130 | 146 | 35 |
| *Passer domesticus* | 0 | 70 | 5 | 0 | 5 | 1 | 19 | 0 | 0 | 0 | 13 | 4 | 46 | 0 | 0 | 0 | 3 | 0 | 8 | 0 | 0 | 0 | 26 | 0 | 0 | 7 | 34 | 32 | 7 | 0 | 12 | 0 | 2 | 1 | 0 | 188 |
| *Passer montanus* | 0 | 0 | 0 | 0 | 0 | 43 | 53 | 0 | 0 | 0 | 0 | 0 | 0 | 0 | 0 | 0 | 0 | 0 | 0 | 0 | 0 | 2 | 0 | 0 | 0 | 0 | 0 | 0 | 0 | 0 | 0 | 34 | 0 | 1 | 0 | 0 |
| *Periparus ater* | 0 | 0 | 0 | 0 | 0 | 3 | 0 | 0 | 3 | 0 | 6 | 0 | 5 | 4 | 0 | 1 | 2 | 0 | 1 | 0 | 0 | 0 | 0 | 1 | 2 | 0 | 1 | 0 | 0 | 4 | 0 | 8 | 0 | 0 | 1 | 0 |
| *Phoenicurus ochruros* | 0 | 0 | 0 | 0 | 0 | 0 | 9 | 0 | 0 | 1 | 0 | 0 | 0 | 0 | 0 | 0 | 0 | 0 | 0 | 0 | 0 | 0 | 0 | 0 | 0 | 0 | 0 | 0 | 0 | 0 | 0 | 0 | 0 | 0 | 0 | 0 |
| *Pica pica* | 0 | 0 | 0 | 0 | 0 | 0 | 0 | 0 | 1 | 1 | 0 | 0 | 0 | 0 | 0 | 0 | 0 | 0 | 0 | 0 | 0 | 0 | 0 | 0 | 0 | 0 | 0 | 0 | 0 | 0 | 0 | 0 | 0 | 0 | 0 | 0 |
| *Picus viridis* | 0 | 0 | 0 | 0 | 0 | 3 | 8 | 0 | 2 | 0 | 1 | 0 | 0 | 0 | 0 | 0 | 1 | 0 | 1 | 1 | 0 | 0 | 0 | 3 | 0 | 0 | 1 | 0 | 0 | 5 | 0 | 9 | 1 | 0 | 1 | 0 |
| *Poecile palustris* | 0 | 0 | 0 | 0 | 0 | 4 | 0 | 0 | 2 | 0 | 5 | 0 | 0 | 25 | 0 | 0 | 9 | 0 | 0 | 0 | 3 | 0 | 0 | 0 | 2 | 0 | 0 | 1 | 0 | 10 | 0 | 10 | 6 | 0 | 2 | 0 |
| *Psittacula krameri* | 0 | 0 | 0 | 0 | 0 | 0 | 1 | 0 | 0 | 0 | 0 | 0 | 0 | 0 | 0 | 0 | 0 | 0 | 0 | 0 | 0 | 0 | 0 | 0 | 0 | 0 | 0 | 0 | 0 | 0 | 0 | 0 | 0 | 0 | 0 | 0 |
| *Pyrrhula pyrrhula* | 0 | 0 | 0 | 0 | 0 | 0 | 2 | 0 | 0 | 0 | 1 | 0 | 0 | 0 | 0 | 0 | 1 | 0 | 0 | 0 | 0 | 0 | 0 | 0 | 0 | 0 | 0 | 0 | 0 | 0 | 0 | 14 | 0 | 0 | 2 | 0 |
| *Regulus regulus* | 0 | 0 | 0 | 0 | 0 | 1 | 0 | 0 | 16 | 2 | 6 | 0 | 17 | 15 | 0 | 0 | 4 | 23 | 0 | 0 | 0 | 0 | 0 | 1 | 5 | 0 | 2 | 0 | 0 | 14 | 0 | 31 | 9 | 6 | 36 | 0 |
| *Sitta europaea* | 2 | 0 | 2 | 6 | 7 | 43 | 47 | 2 | 19 | 12 | 14 | 0 | 4 | 0 | 0 | 0 | 25 | 8 | 15 | 10 | 7 | 5 | 0 | 26 | 16 | 8 | 44 | 52 | 0 | 68 | 0 | 54 | 17 | 16 | 24 | 1 |
| *Streptopelia decaocto* | 0 | 0 | 0 | 0 | 2 | 0 | 2 | 0 | 0 | 0 | 0 | 0 | 16 | 0 | 0 | 0 | 0 | 0 | 1 | 0 | 2 | 0 | 0 | 0 | 6 | 0 | 0 | 11 | 0 | 1 | 0 | 0 | 0 | 0 | 0 | 0 |
| *Troglodytes troglodytes* | 0 | 0 | 1 | 0 | 0 | 0 | 0 | 0 | 0 | 0 | 0 | 0 | 0 | 2 | 0 | 0 | 0 | 0 | 1 | 0 | 0 | 0 | 0 | 2 | 2 | 0 | 0 | 2 | 0 | 3 | 0 | 0 | 2 | 0 | 3 | 0 |
| *Turdus iliacus* | 0 | 0 | 0 | 0 | 0 | 0 | 1 | 0 | 0 | 0 | 0 | 0 | 0 | 0 | 0 | 0 | 44 | 14 | 0 | 0 | 0 | 0 | 0 | 17 | 1 | 0 | 2 | 3 | 0 | 139 | 0 | 0 | 44 | 0 | 71 | 0 |
| *Turdus merula* | 23 | 16 | 4 | 5 | 19 | 22 | 144 | 16 | 64 | 29 | 16 | 2 | 12 | 58 | 6 | 9 | 45 | 14 | 46 | 56 | 3 | 5 | 9 | 33 | 9 | 51 | 129 | 164 | 7 | 77 | 28 | 46 | 84 | 43 | 84 | 20 |
| *Turdus philomelos* | 0 | 0 | 0 | 0 | 0 | 0 | 0 | 0 | 0 | 0 | 0 | 0 | 0 | 0 | 0 | 0 | 1 | 0 | 0 | 0 | 0 | 0 | 1 | 0 | 0 | 0 | 1 | 0 | 0 | 0 | 0 | 0 | 0 | 0 | 0 | 0 |
| *Turdus pilaris* | 0 | 0 | 0 | 0 | 0 | 0 | 3 | 0 | 0 | 0 | 0 | 0 | 1 | 9 | 0 | 0 | 1 | 0 | 2 | 0 | 0 | 1 | 0 | 1 | 0 | 0 | 0 | 0 | 0 | 10 | 0 | 0 | 1 | 0 | 0 | 0 |
| *Turdus viscivorus* | 0 | 0 | 0 | 0 | 0 | 3 | 2 | 1 | 1 | 0 | 0 | 0 | 0 | 1 | 0 | 0 | 5 | 2 | 0 | 0 | 14 | 0 | 0 | 14 | 1 | 0 | 10 | 0 | 0 | 16 | 0 | 4 | 2 | 0 | 15 | 0 |
