# Supplementary material for: Functional diversity of urban bird communities: effects of landscape composition, green space area and vegetation cover
Source: Ecol Evol. 2015 Oct 22;5(22):5230–9. doi: 10.1002/ece3.1778 (PMC6102532; doi:10.1002/ece3.1778)
Supplement: Supplementary file 3 — Table S3. Biological traits of all bird species considered in this study. [file ECE3-5-5230-s003.docx]

**Table S3:** Biological traits of all bird species considered in this study. Foraging substrate: G – ground, F – foliage, B – bark; A – air; foraging method: Gle – gleaning^1^, Pec – pecking^2^, Haw – hawking^3^, Sal – sally^4^, Pro – probing^5^; diet: Bir – birds, Mam – mammals, Fis – fishes, Amp – amphibians/reptiles, Sna – snails, Art – Arthropods, Ann – Annelides, See – Seeds, Fru – Fruits, Car – Carrion. Numbers indicate the extent of usage (0 - not used to 3 - often used).

| **Species** | **Body mass [g]** | **Foraging substrat** | | | | **Foraging method** | | | | | **Diet** | | | | | | | | | |
| --- | --- | --- | --- | --- | --- | --- | --- | --- | --- | --- | --- | --- | --- | --- | --- | --- | --- | --- | --- | --- |
|  |  | **G** | **F** | **B** | **A** | **Gle** | **Pec** | **Haw** | **Sal** | **Pro** | **Bir** | **Mam** | **Fis** | **Amp** | **Sna** | **Art** | **Ann** | **See** | **Fru** | **Car** |
| *Accipiter nisus* | 185.50 | 1 | 0 | 0 | 3 | 0 | 0 | 3 | 0 | 0 | 3 | 1 | 0 | 0 | 0 | 0 | 0 | 0 | 0 | 0 |
| *Aegithalos caudatus* | 7.70 | 1 | 3 | 0 | 1 | 3 | 2 | 0 | 1 | 0 | 0 | 0 | 0 | 0 | 0 | 3 | 0 | 1 | 1 | 0 |
| *Bombycilla garrulus* | 57.80 | 1 | 3 | 0 | 0 | 0 | 3 | 0 | 1 | 0 | 0 | 0 | 0 | 0 | 0 | 1 | 0 | 0 | 3 | 0 |
| *Buteo buteo* | 904.84 | 3 | 0 | 0 | 1 | 0 | 0 | 3 | 0 | 0 | 1 | 3 | 0 | 2 | 0 | 1 | 0 | 0 | 0 | 2 |
| *Carduelis carduelis* | 15.80 | 2 | 3 | 0 | 1 | 1 | 3 | 0 | 1 | 0 | 0 | 0 | 0 | 0 | 0 | 1 | 0 | 3 | 1 | 0 |
| *Carduelis spinus* | 14.00 | 0 | 3 | 0 | 0 | 0 | 3 | 0 | 0 | 0 | 0 | 0 | 0 | 0 | 0 | 1 | 0 | 3 | 0 | 0 |
| *Certhia brachydactyla* | 8.50 | 2 | 0 | 3 | 0 | 3 | 1 | 0 | 0 | 0 | 0 | 0 | 0 | 0 | 2 | 3 | 0 | 1 | 1 | 0 |
| *Certhia familiaris* | 8.50 | 1 | 0 | 3 | 0 | 3 | 1 | 0 | 0 | 0 | 0 | 0 | 0 | 0 | 0 | 3 | 0 | 2 | 0 | 0 |
| *Chloris chloris* | 30.65 | 3 | 3 | 0 | 0 | 0 | 3 | 0 | 0 | 0 | 0 | 0 | 0 | 0 | 0 | 1 | 0 | 3 | 0 | 0 |
| *Coccothraustes coccothraustes* | 55.40 | 2 | 3 | 0 | 0 | 0 | 3 | 0 | 0 | 0 | 0 | 0 | 0 | 0 | 0 | 0 | 0 | 3 | 0 | 0 |
| *Columba livia f. domestica* | 347.00 | 3 | 1 | 0 | 1 | 0 | 3 | 0 | 1 | 0 | 0 | 0 | 0 | 0 | 1 | 1 | 1 | 3 | 1 | 0 |
| *Columba palumbus* | 488.00 | 2 | 3 | 0 | 0 | 0 | 3 | 0 | 0 | 0 | 0 | 0 | 1 | 0 | 1 | 1 | 1 | 3 | 2 | 0 |
| *Corvus corone* | 558.74 | 3 | 2 | 0 | 0 | 0 | 3 | 0 | 0 | 2 | 0 | 2 | 0 | 2 | 2 | 2 | 2 | 3 | 2 | 2 |
| *Corvus frugilegus* | 485.70 | 3 | 1 | 0 | 1 | 0 | 2 | 0 | 0 | 3 | 0 | 1 | 0 | 0 | 1 | 1 | 1 | 3 | 2 | 2 |
| *Corvus monedula* | 244.35 | 3 | 1 | 0 | 2 | 0 | 3 | 0 | 0 | 1 | 0 | 1 | 0 | 0 | 1 | 1 | 1 | 3 | 2 | 2 |
| *Cyanistes caeruleus* | 10.73 | 1 | 3 | 0 | 1 | 2 | 3 | 0 | 1 | 0 | 0 | 0 | 0 | 0 | 0 | 2 | 0 | 3 | 2 | 0 |
| *Dendrocopos major* | 75.55 | 1 | 2 | 3 | 0 | 2 | 3 | 0 | 0 | 1 | 0 | 1 | 0 | 0 | 0 | 2 | 1 | 3 | 1 | 1 |
| *Dendrocopos medius* | 59.00 | 1 | 2 | 3 | 0 | 1 | 2 | 0 | 0 | 3 | 0 | 0 | 0 | 0 | 0 | 2 | 0 | 3 | 1 | 0 |
| *Emberiza citrinella* | 29.80 | 3 | 3 | 0 | 1 | 0 | 3 | 1 | 0 | 0 | 0 | 0 | 0 | 0 | 1 | 1 | 0 | 3 | 0 | 0 |
| *Erithacus rubecula* | 17.97 | 3 | 2 | 0 | 1 | 1 | 3 | 0 | 1 | 0 | 0 | 0 | 3 | 3 | 3 | 3 | 3 | 3 | 2 | 0 |
| *Falco peregrinus* | 697.50 | 0 | 0 | 0 | 3 | 0 | 1 | 3 | 0 | 0 | 3 | 1 | 0 | 1 | 0 | 1 | 0 | 0 | 0 | 1 |
| *Falco tinnunculus* | 209.00 | 3 | 0 | 0 | 2 | 0 | 1 | 3 | 1 | 0 | 3 | 2 | 1 | 1 | 0 | 1 | 1 | 0 | 1 | 1 |
| *Fringilla coelebs* | 23.60 | 3 | 2 | 0 | 1 | 0 | 3 | 0 | 1 | 0 | 0 | 0 | 0 | 0 | 0 | 1 | 0 | 3 | 0 | 0 |
| *Fringilla montifringilla* | 30.25 | 3 | 1 | 0 | 0 | 0 | 3 | 0 | 1 | 0 | 0 | 0 | 0 | 0 | 1 | 2 | 0 | 3 | 0 | 0 |
| *Garrulus glandarius* | 175.00 | 1 | 3 | 1 | 0 | 0 | 3 | 0 | 0 | 2 | 0 | 3 | 0 | 0 | 1 | 1 | 0 | 1 | 3 | 1 |
| *Lophophanes cristatus* | 11.14 | 2 | 3 | 0 | 1 | 3 | 2 | 0 | 1 | 0 | 0 | 0 | 0 | 0 | 0 | 3 | 0 | 2 | 1 | 0 |
| *Nucifraga caryocatactes* | 162.35 | 2 | 3 | 2 | 0 | 0 | 3 | 0 | 0 | 2 | 1 | 1 | 0 | 0 | 0 | 1 | 0 | 3 | 2 | 1 |
| *Parus major* | 19.11 | 3 | 2 | 0 | 1 | 2 | 3 | 0 | 1 | 0 | 1 | 0 | 0 | 0 | 1 | 2 | 0 | 3 | 1 | 0 |
| *Passer domesticus* | 30.40 | 3 | 1 | 0 | 1 | 2 | 3 | 0 | 1 | 0 | 0 | 0 | 1 | 1 | 1 | 1 | 0 | 3 | 1 | 1 |
| *Passer montanus* | 23.00 | 3 | 2 | 0 | 1 | 2 | 3 | 0 | 1 | 0 | 0 | 0 | 0 | 0 | 0 | 2 | 0 | 3 | 1 | 0 |
| *Periparus ater* | 8.98 | 1 | 3 | 1 | 0 | 1 | 3 | 0 | 0 | 1 | 0 | 0 | 0 | 0 | 0 | 2 | 0 | 3 | 0 | 0 |
| *Phoenicurus ochruros* | 17.30 | 3 | 0 | 0 | 2 | 1 | 3 | 0 | 2 | 0 | 0 | 0 | 0 | 0 | 0 | 3 | 0 | 0 | 2 | 0 |
| *Pica pica* | 206.10 | 3 | 1 | 0 | 1 | 0 | 3 | 0 | 1 | 2 | 0 | 3 | 0 | 0 | 2 | 3 | 2 | 2 | 2 | 0 |
| *Picus viridis* | 176.00 | 3 | 0 | 1 | 0 | 1 | 3 | 0 | 0 | 3 | 0 | 0 | 0 | 1 | 1 | 3 | 1 | 1 | 2 | 0 |
| *Poecile palustris* | 11.00 | 1 | 3 | 0 | 1 | 2 | 3 | 0 | 1 | 0 | 0 | 0 | 0 | 0 | 0 | 2 | 0 | 3 | 1 | 0 |
| *Psittacula krameri* | 148.75 | 3 | 1 | 0 | 0 | 0 | 3 | 0 | 0 | 0 | 0 | 0 | 0 | 0 | 0 | 0 | 0 | 3 | 2 | 0 |
| *Pyrrhula pyrrhula* | 32.95 | 2 | 3 | 0 | 1 | 0 | 3 | 0 | 1 | 0 | 0 | 0 | 0 | 0 | 0 | 1 | 0 | 3 | 1 | 0 |
| *Regulus regulus* | 6.80 | 1 | 3 | 0 | 1 | 3 | 0 | 0 | 1 | 1 | 0 | 0 | 0 | 0 | 0 | 3 | 0 | 0 | 0 | 0 |
| *Sitta europaea* | 23.10 | 1 | 2 | 3 | 1 | 1 | 3 | 0 | 1 | 1 | 0 | 0 | 0 | 0 | 0 | 2 | 0 | 3 | 1 | 0 |
| *Streptopelia decaocto* | 210.50 | 3 | 0 | 0 | 1 | 0 | 3 | 0 | 1 | 0 | 0 | 0 | 0 | 0 | 1 | 1 | 0 | 3 | 2 | 0 |
| *Troglodytes troglodytes* | 8.74 | 3 | 1 | 0 | 0 | 3 | 2 | 0 | 0 | 3 | 0 | 0 | 0 | 0 | 0 | 3 | 1 | 1 | 1 | 0 |
| *Turdus iliacus* | 72.80 | 1 | 3 | 0 | 0 | 3 | 2 | 0 | 0 | 0 | 0 | 0 | 0 | 0 | 1 | 1 | 1 | 0 | 3 | 0 |
| *Turdus merula* | 83.35 | 3 | 0 | 0 | 1 | 2 | 3 | 0 | 1 | 1 | 0 | 0 | 1 | 1 | 1 | 2 | 2 | 3 | 2 | 0 |
| *Turdus philomelos* | 70.00 | 3 | 0 | 0 | 0 | 2 | 3 | 0 | 0 | 1 | 0 | 0 | 0 | 0 | 2 | 2 | 2 | 1 | 3 | 0 |
| *Turdus pilaris* | 103.05 | 2 | 3 | 0 | 0 | 3 | 2 | 0 | 0 | 1 | 0 | 0 | 0 | 0 | 1 | 1 | 1 | 1 | 3 | 0 |
| *Turdus viscivorus* | 109.40 | 2 | 3 | 0 | 0 | 3 | 2 | 0 | 0 | 1 | 0 | 0 | 0 | 0 | 1 | 1 | 2 | 1 | 3 | 0 |

^1^ picking food items from a substrate without manipulation of the surface; ^2^ removing some of the exterior of the substrate by using the bill; ^3^ attacking in continuous flight; ^4^ flying from a perch to attack a food item and then return to the perch;
^5^ inserting the bill in holes of a firm substrate or directly into softer substrate to capture hidden food
