# Supplementary material for: Functional diversity of urban bird communities: effects of landscape composition, green space area and vegetation cover
Source: Ecol Evol. 2015 Oct 22;5(22):5230–9. doi: 10.1002/ece3.1778 (PMC6102532; doi:10.1002/ece3.1778)
Supplement: Supplementary file 4 — Table S4. Correlation coefficients of Pearson correlations including the park variables park area (log transformed) and canopy heterogeneity as well as the landscape variables natural green space, man‐made green space, forest and sealed area, describing the urban matrix surrounding each city park, are listed. Significant correlations (P < 0.05) are printed in bold. [file ECE3-5-5230-s004.docx]

**Table S4:** Correlation coefficients of Pearson correlations including the park variables park area (log transformed) and canopy heterogeneity as well as the landscape variables natural green space, man-made green space, forest and sealed area, describing the urban matrix surrounding each city park, are listed. Significant correlations (*p* < 0.05) are printed in bold.

|  | **Park area (m^2^)** | **Canopy heterogeneity (m/ m^2^)** | **Natural green space (m^2^)** | **Man-made green space (m^2^)** | **Sealed area (m^2^)** | **Forest (m^2^)** |
| --- | --- | --- | --- | --- | --- | --- |
| **Park area (m^2^)** | 1.00 | -0.26 | 0.27 | 0.14 | **-0.54** | 0.01 |
| **Canopy heterogeneity (m/ m^2^)** | -0.26 | 1.00 | -0.28 | -0.22 | **0.34** | -0.18 |
| **Natural green space (m^2^)** | 0.27 | -0.28 | 1.00 | 0.20 | **-0.50** | **0.39** |
| **Man-made green space (m^2^)** | 0.14 | -0.22 | 0.20 | 1.00 | **-0.76** | 0.25 |
| **Sealed area (m^2^)** | **-0.54** | **0.34** | **-0.50** | **-0.76** | 1.00 | -0.32 |
| **Forest (m^2^)** | 0.01 | -0.18 | **0.39** | 0.25 | -0.32 | 1.00 |
